# Dental Patents

**Published:** 1853-04

**Authors:** A. Hill

**Affiliations:** Norwalk, Ct.


### ARTICLE V.

*Dental Patents.* By A. HILL, D. D. S., Norwalk, Ct.

OF late there has been considerable discussion upon the subject of dental patents, and quite a diversity of opinions expressed with reference thereto. Some of our brethren whose zeal seems to have got the mastery over their judgments, have come down upon the subject in a manner by no means calculated to win our suffrages, or impress our understanding with a conviction of the correctness of their reasoning.

Grandiloquent declamation—or wholesale denunciation of those who are opposed to us, will not always pass for logic.

Neither will all the changes, that may be rung on the various epithets in common use, among a certain class of writers, be substituted by thinking men, for legitimate deduction.

It shall be our business to clear away the rubbish, and extraneous matter by which the subject is surrounded, as far as may be, and offer a few considerations which to our own mind seems to be of importance touching the matter in question.

It is confessed on all hands, that a dentist or physician, has a *legal* right, to secure letters patent for any article, or invention that his ingenuity or labor may devise, this, then, is not a bone of contention. We think that the *moral* right to a patent must also be conceded, where no moral principle is sacrificed in its exercise. For a patent in itself can no more be considered an immorality, than any other species of property, that is held by a just and lawful title. As well might we say, that *wealth* is an immorality—or talents an immorality, as to say that a *patent right* is nevertheless a patent *wrong*, and that of necessity.

Property, honestly secured, is a *right*—a legal right, a moral right, and may be a great blessing, when properly used. This, too, we think is not the question in dispute.

But the *professional* right, aye, here is the rub, we are such sticklers for conventionalities—for time honored customs—and even for hoary errors, that we can scarcely allow this matter to be called in question, without becoming venomous as serpents. But let us have patience—may be, we shall get at the truth after all.

Let us look this thing full in the face, and see what we can make of it. We address ourselves, then, to the disputed point, which comes to this; we may not do professionally, what we may do legally, and of moral right.

There is not, necessarily, any thing dishonest in a patent, it is only "*dishonorable*," and it only becomes dishonorable, by being a breach of conventionality. And it is only a breach of conventionality, when it violates constitutional laws, or usages. We are speaking now as to the necessity of the case.

We grant that a thing—an act, or a patent *may* be, not only dishonorable, but absolutely dishonest, where it deprives another member of the same profession of his just rights, and the community of those advantages, which would accrue to them, if no such thing existed. But no such *necessity* attaches itself to the subject under consideration. We therefore cast it aside, as irrelevant.

The subject becomes exceedingly simple, when divested of its surplus drapery, and vague generalities. But it is not often discussed in this way, or even in a spirit favorable to the development of truth.

When an individual is admitted to fellowship in any society, having a constitution or by-laws, or both, it is expected of him, that he will conform both to their letter and spirit. If one of those articles forbid the procurement of a patent, and he becomes a patentee, it is clear, that he has violated the principles of fellowship in such society. His offence is a conventional one, and under certain circumstances might be characterized as an immorality. But if there be no article in the constitution, or by-laws, or any vote or resolution, passed by the society, which prohibits the right of any member to secure letters patent, has he then violated the terms of the compact? We think not.

We will go farther and say, that if it can be shown, that immemorial custom, or usage in the society of which he is a member has reprobated the principle of patents, he has committed an offence, for which due atonement should be made. Can this be said of our profession, or any society of practitioners, connected with it? If so, we stand convicted, if not, we are clear.

With respect to the medical profession proper, we are aware of a strong feeling against the principle of patents, in the profession, and we have been taught to regard it, as dishonorable for a medical practitioner to become a patentee, in things pertaining to practice. Although the catalogue of M. D's. guilty of this offence is really formidable.

But what is the process of reasoning by which we reach the conclusion, that it is dishonorable in a medical or dental practitioner to procure letters patent? If it be the mode suggested above, then let us put it on that ground, and that alone. Call it simply, a conventional sin, and deal with the offender accordingly. If it be dishonorable in the sense of an immorality, then reprobate it as such. If it be dishonorable in the sense of *quackery*, prove it to the world, and brand the offender as a "quack." But what is this thing you term "quackery?" If we mistake not, it is a term susceptible of various meaning, and very comprehensive significance. And it is, moreover, in the mouths of many persons a most convenient epithet. It is to some what the cry of "*stop thief*" is to the rogue. It is to others an easy vehicle of calumniation and abuse. But heaven save the mark—where it is justly and strictly applied, to the extent which is merited. We can only say, "let him who is without sin cast the first stone." We have consulted Noah Webster upon the subject, but we will not trouble the reader with quotations.

It will answer our purpose to remark, that patentees are not the only "quacks" in this wide world of ours, and that those who are conscious of this guilt, are generally most fond of bestowing the epithet on others. Like some quarrelsome darkies we have seen before this, who, when they become angry at each other, and wish to heap up in one word, the utmost contempt, and scorn, call each other "*niggers.*"

But to the subject.

Let the reader bear in mind, that it is not our present business to vindicate the *conduct* of patentees, nor to show that they never abuse their privileges. Neither are we contending for the propriety of patents in all cases; but for the *right.* Legal, moral and professional.

It will not do to say, that patentees are the most "*avaricious*" of mortals, that they are ready "to thrust their lucre-loving hands into the pockets" of honest people. This is not true. Would to heaven it were true, that they, and they *only* were guilty of the sin of avarice or covetousness. Condemn this thing as heartily, and as much as you please, but do not make it exclusively the sin of patentees.

If you say the profession is too "holy," too noble, too exalted a calling, to make it the means or source of gain, do you not necessarily condemn those who have amassed the most splendid fortunes, in simply professional pursuits? Do you not discountenance the legitimate demand for a fee in any case? And especially such exorbitant fees, as some of the most eminent practitioners require?

Why not put the support of the physician and dentist on the same footing with that of the clergy, viz. the voluntary contributions of the people? and thus make their services free, to the poor and abject portion of the community.

Men's genius, and talents are various, and run in different channels, on what principle is the labor and genius of one man to be appropriated to the use of the public without reward, and the other extravagantly paid for his services. How is it that the man who toils in his laboratory, and taxes his energies by night and by day, for years together, and expends all his time and money to invent and perfect some useful compound or machinery, is required to yield his rights to the noisy requisitions of the public, or profession to which he belongs; while another man becomes eminently skillful in the use of these very compounds, or instruments, is allowed to acquire an immense fortune by placing very high terms, as the only condition upon which his skill becomes available.

For illustration, let us suppose a case.

Dr. Banning turns his attention, and devotes his time to the perfection of a "body-brace," or abdominal supporter, and in order to indemnify himself for his time, and labor, and expense, he takes out letters patent, and sells his instruments at a price that he deems a fair compensation. Now Dr. Nameless would not be guilty of such a thing, as taking out a patent, but he is eminently skillful in surgery, and must be paid for the exercise of that skill, therefore he expects a fee, which excludes by far the largest portion of the community from the benefit of his services, and according to this system of ethics, Dr. Nameless is an *honorable*, *high minded* and *chivalrous* fellow, while Dr. Banning, is a mean contemptible quack that ought to receive no fellowship from an honorable profession.

Is that it?

Again, here is Dr. J. Allen, who has toiled most laboriously in his laboratory—has expended hundreds of dollars, and years of time, to complete his great improvement in mechanical dentistry, and has secured his interest by a patent. Now what shall be done with him? (Bear in mind, we are not now to inquire into the validity of his patent, or the priority of invention, but presuming every thing right and fair on this score.) What say these chivalrous, whole-soul practitioners of dentistry? How shall he be indemnified? O says Keyes, "the day has arrived, when those who love their profession, and would lend their aid to elevate and improve it, should eschew the patent, as they would dishonesty, should hold themselves aloof from every species of quackery trusting wholly to moral and professional merit for success." He must not get a patent, because he thereby "violates the ethics" of his profession and "because he owes to the profession the best energies of his mind, and the benefit of his experience."

But we ask again, how shall he be indemnified? Can a man live on wind, or glory, which is much the same. Does he owe nothing to his family? May he not "provide for his own and especially those of his own household?"

And what is this thing you call "moral ethics?" And by what code of "moral ethics" do you propose to appropriate this man's labors to your own, and the public use, without indemnity? If any member of our profession, by virtue of superior skill, and an eminent position in society, can command, and do receive from *ten* to *thirty* dollars for filling a single tooth, by what code of morals shall he be allowed to receive what some would deem enormous fees, while this very circumstance must necessarily deprive the masses of the community of the benefit of his services, while the other man toils on, purely for the public good?

But this does not "detract from the dignity and respectability of the family to which he is attached, the circle in which he moves, or the community in which he lives." It rather increases the "respectability" of the concern. But how does it comport with the character of him who "goes forth full of charity and love, with the exalted hope—the high resolve, to do what he can to alleviate the sufferings of his fellow-men?" How comport with him, who is "clearly above the base and sordid things of earth?" and so extendedly engaged in the exercise of charity which so elevates and adorns our natures?"

The great error here, most obviously lies in assuming, that the man who secures a patent, is *necessarily*, supremely selfish and *grasping*. This, however, remains to be proved.

Would it not be pertinent to the subject to inquire, how many who are now engaged in the practice of our profession, would continue in it, if the simple idea of *gain*—of *money-making*, were at once and forever dissevered from it? How many of the few who command extraordinary prices would continue in the profession, if the more moderate compensation of the many, should become the inevitable standard?

But suppose the talents of some member of the profession should lead him into a series of experiments involving time, labor and expense, and eventuates in the discovery of a process by which his professional brethren can be greatly advantaged, and immense good to mankind result from it: meantime, in perfecting and bringing his discovery before the world, his means are all exhausted—his family destitute, and he greatly embarrassed with debt, and that debt incurred in prosecuting his experiments. But with the devotion peculiar to this class of persons he continues, and by "hook or by crook" he completes the invention. The thing is done, but he is bankrupt. What is his duty, under the circumstances?

O, says Keyes, "he who resorts to such a course (i. e. to procure a patent) to secure notoriety and gain, merits the opprobrium, and obloquy of his brethren, and should have the hand of fellowship withheld, and treated as an outlaw." This would be "befouling yourself with things unclean, or habits unholy." This would be "thrusting his polluted lucre-loving hands into the pockets of the poor, the honest, the ignorant, the sick and the dying."

But what shall he do? He must elevate himself above all these "sordid" considerations, and upon the "noble altar of the public good," consent to sacrifice the happiness of his innocent family. Ours is a "liberal profession," and we must show our liberality by giving our time, talents and services to those best able to pay for them, i. e. the profession. For "the day has arrived, when those who love their profession and would lend their aid to elevate and improve it, should eschew the patent, as they would dishonesty; should hold themselves aloof from every species of quackery, trusting wholly to moral and professional merit for success."

It does not comport with the lofty character of the members of our profession, to be inquiring "what they shall eat," &c., such things are decidedly vulgar. You must give it to the profession, or "be treated as an outlaw."

But replies the unfortunate inventor—my time, money and labor belong to my family, at least in part, and duty requires of me to look after their interest.

"But you will lose all the glory of a noble act."

Glory will not buy bread. Glory will not cancel my obligations. Glory will not educate my children—and, beside, if you are such a stickler for *glory*, *dignity* and *usefulness*, are you not willing to share it with me? Why not then come forward, as a profession, each one contributing according to his ability, to enable me without loss or detriment to give it to the world? This would, indeed, be something worth talking about. This would be *chivalrous*, *glorious*, *liberal.*

It is in this view of the matter, that we discover that this vaunted system of ethics is completely crazy. And that instead of philanthropy and benevolence, and noble generosity, selfishness itself, like a serpent, lies concealed in all this high-sounding verbiage. For, in effect, it demands our time, labor and money, without compensation. It virtually says, if you will give it up, well and good; if not, we will oppose you at every step; we will blacken your professional character and reputation at every opportunity; we will brand you as a quack, and treat you as an "outlaw." We will invalidate your claim, if possible, oppose the sale of your *rights*, and by a system of piracy and a perpetual guerilla warfare, we will ruin you.

And so unscrupulous is this opposition, and to such an extent has it been carried, that we frankly confess that dental patents in these days are of little worth. But whether valuable or not, the morality of such conduct, on the part of many who oppose them, is, to our mind, exceedingly questionable.

The man who writes a book, or treatise, can secure himself by means of a *copy-right.* This is *honorable*, even praiseworthy. But this man, instead of writing a book, has worked out a process, or invented an instrument, and where is his protection? No where, except in a patent, and that is disreputable. Here are moral ethics with a vengeance! But, it is contended, that *copy*-right is widely different from a *patent*-right, and by no means analogous. Let not the candid reader be misled by mere verbiage. He will be surprised at the similarity, the identity of principle, if he will attempt the analogy.

Here, then, is our position, and here will we entrench ourselves. This *pet*-lamb of our profession must die with the *goat.* This favorite child has no more legitimacy than this much abused and kicked-about bantling. Gentlemen may nettle, but we have the hook where it is secure, and our fish must come to land. We are by no means ignorant of the method of argumentation upon this subject. But still, we confidently count upon our game. For a blow at the one, is a blow at the other. These Siamese twins cannot be separated without risk. They were born of the same parentage—nursed by the same hand, and the throbbing heart of the one, sends its pulsations through the arteries of the other.

And here it is that we complain of injustice, of inequality. Here, we enter our solemn protest against hoary usage, and obnoxious farvoritism. Here, we learn to appreciate the grandiloquent declamation, and elegant emptiness, of much of this "sound and fury," which either destroys *copy*-rights, or "signifies nothing," when directed against *patent*-rights.

Let us see how they look side by side where they properly belong. The one is an *exclusive* right, so is the other. The one is a *monopoly*, so is the other. The one costs time and labor, so does the other. The one may be valuable or otherwise, so may the other. There is no secret about either. Every plea for one, is an argument for the other, and so on *ad libitum.*

Thomas B. Macauley, in one of his speeches before the British Parliament, holds the following language in reference to this matter: "Copy-right is monopoly, and produces all the effects which the general voice of mankind attributes to monopoly." * * * "It is good that authors should be remunerated, and the least exceptionable way of remunerating them is by a monopoly." * * * "The principle of copy-right is this: It is a tax on readers, for the purpose of giving a bounty to writers."

But, replies Prof. Handy, "what each one can add to the common stock, it is considered not only that he receives out of that common stock in return a full equivalent, but further, a measure fully heaped up and running over; and that mind, it is believed, must be possessed of no small share of vanity, to think that all other minds have contributed nothing to the common stock, and that from this universal poverty of mental contribution, this alone luminary, receiving nothing in return, modestly comes out and claims a patent (copy-right) for extra contributions of services rendered."

Now we humbly submit, that the foregoing aims a vital blow, against *copy*, as well as *patent*-rights. Has the man who writes a book the vanity to suppose that he can add anything to the common stock? And what has he, that he did not receive? An editor, (an able one too,) in discussing in his paper the copy-right question, asks the following: "Can we be wrong in regarding the naked issue of honesty against knavery, as forming the base of the copy-right question?"

And if the principle involved is the same as the patent principle, can we be wrong in regarding the naked issue of honesty against knavery as forming the base of the patent-right question? There is perhaps this difference between the two. While the rights of *inventors* are limited to a few years, the rights of *authors* have no such limitation.

It may be said, that many of the patents which are procured in these days, cover the most worthless pretensions; or, in other words, the patent article has no value, no real worth, and amounts to nothing. This is doubtless very true, but what then? If the patent covers no valuable improvement, it can certainly work no injury to the profession; for it is no loss to be without it; and the patentee is the worse for his labor and expense. But is not all this true of the copy-right? How many worthless volumes fall dead from the press? How many come into the world still-born, and never pay the expense of publication?

In the July number of the American Journal of Dental Science, for 1852, Prof. Handy bases an argument against patents in medicine, on the ground of a "general feeling," both professional and non-professional, against them. He says, "there is no legal difficulty in the way; but only the general feeling, both professional and unprofessional, which was (wars?) against it, and it is just this feeling which seems to form the reason why the opinion is so prevalent that patents in all such cases are wrong. Now, if such feeling as this were only occasional, and occurred but in few cases, it might be thought of but little value, and seem scarcely worthy of notice; but when, on the contrary, such feeling becomes universal; when it is found in the savage, as well as civilized man; in the ignorant, as well as the informed, the inference seems fairly to force itself that such feeling is fundamental to our nature, of binding authority, and enforcing a moral obligation, which every one feels as instinctive to his very being." "Now, if this be true, (he says,) the common opinion of mankind is against all patenting whatever in medicine," &c.

"*If this be true*;" but we ask most respectfully, is this true? If the fact be, as stated by Prof. Handy, we admit the force of his conclusions; otherwise the argument fails. We judge that the feeling is neither so *general* nor *instructive* as to give much force to the opinions of a "savage" on the subject. And, so far as we are informed upon the matter, our inference is quite different from the one drawn by Prof. Handy. And it would seem that the very general use and sale of patent medicines and secret compounds in the most enlightened and refined communities, was proof incontestable of a *general* feeling in their favor. At all events, it must weaken the force of the argument drawn from the assumed fact, that the general voice of mankind is instinctively against patents in medicine.

The following proposition we do not choose to dispute, viz. "That he who would obtain an exclusive privilege by patents to save men's lives, and that only such as would choose to pay for, and obtain the healing virtues of such patent, must be doomed to die, would at once be pronounced a very monster of avarice and cruelty; a being in human shape, but thoroughly destitute of all the feelings of a common humanity, as well as of common decency."

But is it just—is it ingenuous, to draw a sweeping conclusion against *all* patents, even in medicine, from a supposable case like the above? Do we not generally reason from false premises, on this subject, and are not such deductions illogical?

Granted—that "the healing of the sick, is of such a high and holy character, and involves such weighty responsibilities as not, for a moment, to be put in contrast with, or placed by the side of, simply dollars and cents." * * * *

Granted—that "the art of healing has more than once been styled a divine art—one not to be acted out, and measured by pecuniary profit—but one to be free as the air we breathe," &c.

What then? If this proves any thing, does it not prove that it is absolutely degrading to set a *price*, a *fee*, upon medical services? Does it not prove that the services of a physician ought to be placed upon the same ground as those of the clergy? Is it right for a man to dole out or dose out his skill in saving either human souls, or human bodies, at *one* or *five* dollars per dose? Why should dollars and cents stand in the way of human life, or human salvation? And yet it is manifestly true that they do.

One of two things must be true—if the above proposition is valid, either the whole system of medical compensation is wrong and should be condemned; or else the right to a patent in medicine or surgery is justifiable. This broad sword has two edges, and the one is as sharp and terrible as the other.

Does the price of a patent, or the right to use it, stand in the way of human life? so do the price and advantages of superior medical skill, and the exorbitant fees of some of our most eminent physicians and surgeons, give them a monopoly a thousand times more fearful and detrimental to human life and comfort than any medical or dental patents ever can do. We insist upon it, that this subject has been discussed in a one-sided and partial manner; and that the arguments adduced against patents, either prove too much, or they are unjustly restricted in their consequences.

We are willing to admit great abuses in the patent system, but let us wisely discriminate in matters of principle.

But—"The medical profession is regarded as a common profession—as one great brotherhood for the common good, consequently having one common interest. And thus, by the common contract necessarily having a common share to the accumulative property belonging to the common stock."

Is not this rather specious than otherwise? Its poetry is certainly fine, but for sober prose it is *quite* specious.

Surely Prof. Handy cannot mean to convey the impression, that by virtue of his, or other connection with the profession of medicine, that therefore he is entitled to share in the wealth of his more fortunate brethren. No! he only means to convey the impression that we are entitled to *know* as much as any of the brotherhood, if our small craniums can stow it all away.

The argument that no one can possibly add to the common stock of human knowledge any more than he has received, only seems to render the subject more difficult, and complex, as it is here applied.

For this also, if it proves anything, can be successfully turned against the position of those who oppose the views for which we contend. For where is there a human being upon the face of the earth, be the sum of his knowledge much or little, that is not indebted to society for all that he knows, and for all that he has?

This argument is transcendental—it destroys *all* property distinctions, of whatsoever kind. It is clearly a plea, for not merely a community of interest, but a community of rights. And if pushed to its consequences *Fourierism* itself could demand nothing more.

We see then that the argument is defective simply because 1st, the facts are *assumed* without proof, and 2nd, because the premises being defective, the deductions are necessarily so. The argument also which Prof. Handy draws from the relation of *splints, trusses,* &c., to human life and health, we think is equally defective. Because, 1st, if carried to its consequences, it would inevitably overthrow the whole patent system altogether. 2nd, it is based upon the assumption that a patent right *necessarily* stands in the way of human life, and health, which remains to be proved.

There are a thousand various objects concerning the right to patent which, no question has ever been raised that sustains a relationship to human life and health, as intimate and special, as the relation of splints to broken limbs, trusses to the cure of hernia, &c. For instance—

Food is essential to life, &c. Now that which effects the price of food, is related to human life. A patent *plough—harvester* —a *mill* for grinding grain—conveyances to market, &c., may all be connected with human life in the matter of food. Will none of these things admit of a patent?

Again. Clothing is essential to human life and comfort, and cheap clothing is connected in a variety of ways, with patent machinery, &c. Where shall the argument end?

But our article is of sufficient length, and should be concluded. We have intended to preserve a respectful bearing toward our brethren whose views may differ from our own, and we should regret to know that any just cause of offence should be found in anything we have written upon the subject.

For the respectful manner in which Prof. Handy has treated the subject, he has our high regard.

He has very justly remarked, that the subject is one of very great interest. "That it is a subject admitted to be of some difficulty, about which honest minds may differ." We do not set ourselves for the defence of all its abuses, but merely inquire as to its *right* and *propriety* in certain cases, and unless we have entirely misapprehended the whole matter, we think we have a right to complain of the offensive personalities—the invidious comparisons, and abusive epithets, in which *some* who have written upon this subject, have so freely indulged.

We are not yet quite willing to be treated as an "*outlaw*," or branded as a *quack*. We may yet see cause to change our views upon the subject of patents, but such kind of treatment will not hasten our conversion, or conciliate our feelings.

May we not therefore invoke a nobler, and more generous spirit, in the discussion of this subject.

### ARTICLE VI.

*American Dental Patents.* By GEO. W. KENDALL, Cincinnati, Ohio.

